# Molecular Physiological Evidence for the Role of Na^+^-Cl^−^ Co-Transporter in Branchial Na^+^ Uptake in Freshwater Teleosts

**DOI:** 10.3390/ijms24076597

**Published:** 2023-04-01

**Authors:** Shang-Wu Shih, Jia-Jiun Yan, Shao-Wei Lu, Ya-Ting Chuang, How-Wei Lin, Ming-Yi Chou, Pung-Pung Hwang

**Affiliations:** 1 Institute of Cellular and Organismic Biology, Academia Sinica, Taipei 115201, Taiwan; frank4xx36@gmail.com (S.-W.S.);; 2 Department of Life Science, National Taiwan University, Taipei 106319, Taiwan; 3 Department of Bioscience and Biotechnology, National Taiwan Ocean University, Keelung 202301, Taiwan

**Keywords:** Ncc, Nhe, Na^+^ uptake, in vivo functional assay, adult, gills, zebrafish

## Abstract

The gills are the major organ for Na^+^ uptake in teleosts. It was proposed that freshwater (FW) teleosts adopt Na^+^/H^+^ exchanger 3 (Nhe3) as the primary transporter for Na^+^ uptake and Na^+^-Cl^−^ co-transporter (Ncc) as the backup transporter. However, convincing molecular physiological evidence to support the role of Ncc in branchial Na^+^ uptake is still lacking due to the limitations of functional assays in the gills. Thus, this study aimed to reveal the role of branchial Ncc in Na^+^ uptake with an in vivo detection platform (scanning ion-selective electrode technique, SIET) that has been recently established in fish gills. First, we identified that Ncc2-expressing cells in zebrafish gills are a specific subtype of ionocyte (NCC ionocytes) by using single-cell transcriptome analysis and immunofluorescence. After a long-term low-Na^+^ FW exposure, zebrafish increased branchial Ncc2 expression and the number of NCC ionocytes and enhanced gill Na^+^ uptake capacity. Pharmacological treatments further suggested that Na^+^ is indeed taken up by Ncc, in addition to Nhe, in the gills. These findings reveal the uptake roles of both branchial Ncc and Nhe under FW and shed light on osmoregulatory physiology in adult fish.

## 1. Introduction

Osmoregulatory animals must control the gain and loss of Na^+^ for body fluid osmotic and ionic homeostasis. Vertebrates living in seawater (SW), freshwater (FW), and land have evolved different ionoregulatory traits for maintaining internal Na^+^ balance. The gills are the dominant ionoregulatory organs for Na^+^ transport in adult teleosts, but this process is mediated by the skin of embryos/larvae before gill formation. These organs contain a high density of ionocytes (mitochondria-rich cells that highly express basolateral Na^+^/K^+^ ATPase, Nka), which are the major sites for teleost ion regulation [1,2,3]. In FW teleosts, due to a huge ion gradient between hypotonic FW and body fluids, the active uptake of Na^+^ is required for maintaining the physiological state and survival in order to compensate for the passive loss of Na^+^ to the surrounding water. To date, three models have been proposed for taking up Na^+^ from FW by the gill/skin ionocytes: (1) apical Na^+^/H^+^ exchanger (Nhe)-mediated, (2) apical vascular-type H^+^ ATPase (VHa)-driven acid-sensing ion channel (Asic)-mediated, and (3) apical Na^+^-Cl^−^ co-transporter (Ncc)-mediated pathways [4,5,6,7,8,9]. That said, it appears that the VHa-driven Asic model can be eliminated [9] because most FW teleosts do not apically express VHa in the ionocytes [10] and Asic exhibits an unfavorable gating property under FW [11,12,13]. Therefore, the Nhe and Ncc models have likely become the mainstream concepts based on molecular/cellular/physiological evidence from several FW teleost species [8,9,14].

Nhe3 and Ncc are thought to be expressed in different ionocyte subtypes and contribute to Na^+^ uptake with a dominate and a minor (or backup) role, respectively, in FW teleosts [15,16,17,18,19]. Functional analyses on the larval skin of zebrafish provided direct evidence to support their distinct contributions to Na^+^ uptake. In larval zebrafish, VHa-rich (HR) ionocytes (Nhe3b-expressing cells), instead of Ncc2-expressing ionocytes (NCC ionocytes) or other cell types, could visibly accumulate a fluorescent Na^+^ dye (sodium green), which could be severely reduced by an Nhe inhibitor (5-(N-ethyl-N-isopropyl)-amiloride, EIPA) [20]; nevertheless, the uptake functions of Na^+^ and Cl^−^ via Ncc were proven in larval skin treated with an Ncc inhibitor (metolazone) by Wang et al. [21]. Furthermore, zebrafish Ncc2 was revealed to compensate for Na^+^ uptake by increasing its mRNA expression and the number of Ncc2-expressing ionocytes in the larval skin after Nhe3b knockdown [22]. These pieces of evidence suggest a minor/backup involvement of Ncc in teleost Na^+^ uptake. However, some findings have challenged and questioned the role of Ncc. Acclimation to low-Na^+^ FW was not necessary to upregulate Ncc mRNA expression in both the adult gills and whole larvae [18,21,23,24,25]. Neither metolazone treatment nor incubation of Cl^−^-free FW reduced the whole-body Na^+^ influxes in adult zebrafish [7], which appears to conflict with the study by Wang et al. [21]. A knockout (KO) experiment in zebrafish larvae challenged the role of Ncc in Na^+^ uptake in *nhe3b* mutants with an unchanged mRNA expression of Ncc and unaffected whole-body Na^+^ influxes by treating with metolazone or Cl^−^-free FW [24]; however, this study seemed to neglect that mRNA expression analyses of whole embryos/larvae may not reflect a real response in skin ionocytes. Without further investigation on the changes in protein levels or differentiation of ionocytes, evidence on transcriptional expression largely limits our understanding on Ncc regulation. On the other hand, there has been no study to directly detect Na^+^ uptake activity in adult gills thus far. As is broadly known, the gills presumably contain many more ionocytes than larvae, which is more reliable for use in analyzing ion-transporting functions. Compared to the whole-body detection of adult fish, real-time detection on the gills can easily be observed by voltage changes (by electrophysiological tools) and directly reflect the responses of ionocytes/ion transporters. Detection on the gills would be necessary to clarify the function of Ncc. As such, a comprehensive investigation on the adult fish gills by a variety of approaches is needed to revisit the contribution of Ncc to body fluid Na^+^ homeostasis in FW teleosts.

To address these issues and clarify the debates above-mentioned, the present study adopted different approaches including single-cell RNA sequencing (scRNA-Seq), electrophysiology (scanning ion-selective electrode technique, SIET), pharmacology, and molecular/cellular biology to ask the following specific questions: (1) Can a distinct subtype of ionocytes (NCC ionocytes) be identified in zebrafish gills by scRNA-Seq? (2) Do zebrafish gills upregulate Na^+^ uptake capacity in response to low-Na^+^ FW? (3) Is Ncc upregulated at the level of mRNA, protein, and/or ionocyte after acclimation to a low-Na^+^ FW? Our results provide convincing and comprehensive evidence to demonstrate the role of Ncc in the gill Na^+^ uptake mechanisms of FW teleosts, providing new insights into fish gill osmoregulation.

## 2. Results

### 2.1. Branchial NCC Ionocytes Are a Specific Subtype of Ionocytes

**Figure 1:**
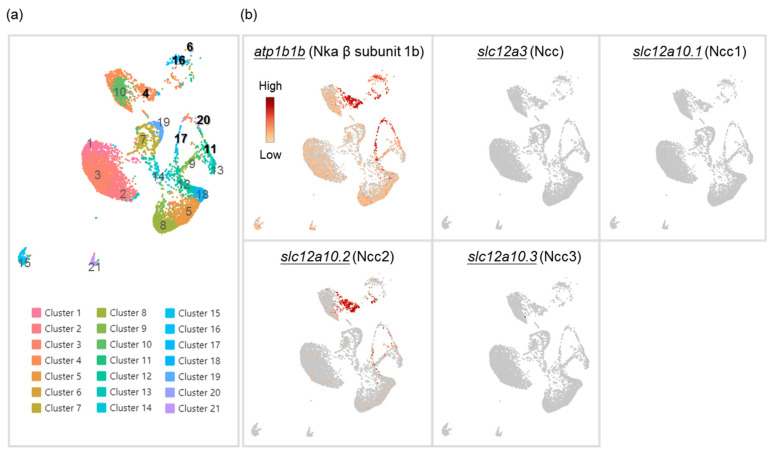
Cell clustering of gill cells by single-cell RNA sequencing (scRNA-Seq) analysis in adult zebrafish. scRNA-Seq shows 21 cell clusters in the gill on the UMAP (**a**). The distribution and expression of *atp1b1b* (Nka β subunit 1b), *slc12a3* (Ncc), *slc12a10.1* (Ncc1), *slc12a10.2* (Ncc2), and *slc12a10.3* (Ncc3) are shown on UMAP in the zebrafish gill cells (**b**).

An ionocyte-like expression pattern of Ncc2-expressing cells was revealed in the gills of zebrafish [22]; however, whether they are a distinct subtype of ionocytes has not been clearly assessed. Therefore, we first conducted single-cell RNA sequencing (scRNA-Seq) to cluster gill cells based on the expression profile of each cell (Figure 1a). We found a higher expression of *atp1b1b* (Nka β subunit 1b, a marker for all ionocytes in zebrafish [26,27]) in Clusters 4, 6, 11, 16, 17, and 20. The mRNA of *slc12a10.2* (Ncc2) could be highly detected in the gills and was mostly expressed in Cluster 4 (Figure 1b), while the mRNA of other Ncc paralogs (*slc12a3*, *slc12a10.1*, and *slc12a10.3*) could not, which suggests that Cluster 4 is a subtype of ionocytes that express Ncc2 (NCC ionocytes). Meanwhile, Clusters 11, 17, and 20 were identified as VHa-rich (HR) ionocytes (expressing VHa and Nhe3b) (Figure 1a and Figure S1a). Clusters 6 and 16 were identified as epithelial Ca^2+^ channel (Ecac)-expressing ionocytes, also called Nka-rich (NaR) ionocytes (specifically stained by an Nka α subunit antibody [1,28,29]) (Figure 1a and Figure S1a). As such, these findings suggest that NCC ionocytes are distinguishable from the other two ionocyte subtypes. We further found that these three distinct subtypes of ionocytes expressed different paralogs of the Nka α1 subunits, respectively (Figure 1a,b and Figure S1a). The HR ionocytes expressed *atp1a1a.2*, *atp1a1a.3*, and *atp1a1a.4*. Ecac-expressing ionocytes expressed *atp1a1a.1*. NCC ionocytes expressed *atp1a1a.2* and *atp1a1a.3*. These findings are briefly summarized in Figure S1b.

**Figure 2:**
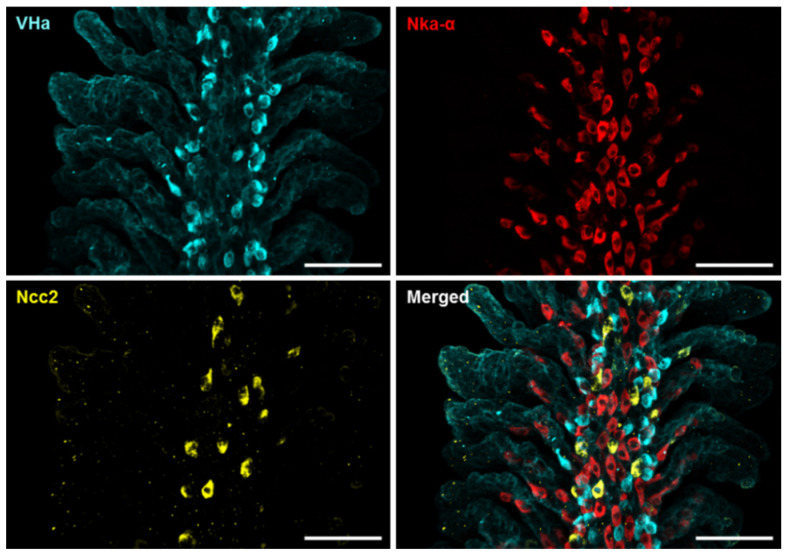
Localization of the three subtypes of ionocytes in the gills of adult zebrafish. Whole-mount immunofluorescence (IF) of the gills was used to reveal the expression patterns of VHa-rich (HR) ionocytes, Nka-rich (NaR) ionocytes, and NCC ionocytes with antibodies against vascular-type H^+^ ATPase (VHa), Nka α subunit (Nka-α), and Ncc2, respectively. A different angle of this image sample is shown in Figure S2. Scale bar, 50 μm.

To confirm the scRNA-Seq data, whole-mount immunofluorescence (IF) of the zebrafish gills was performed using three different antibodies against Ncc2, the Nka α subunit, and VHa, respectively, to identify the three ionocyte subtypes above-mentioned. Triple IF revealed that the signals of Ncc2, the Nka α subunit, and VHa were not co-localized (Figure 2 and Figure S2), suggesting that the NCC ionocytes, NaR ionocytes (Ecac-expressing ionocytes), and HR ionocytes are different subtypes. The specificity of the Ncc2 antibody was verified by peptide blocking and is shown in Figure S3.

### 2.2. Low-Na^+^ FW Increased the Branchial Protein Expression of Ncc2 but Did Not Affect Its mRNA Expression

**Figure 3:**
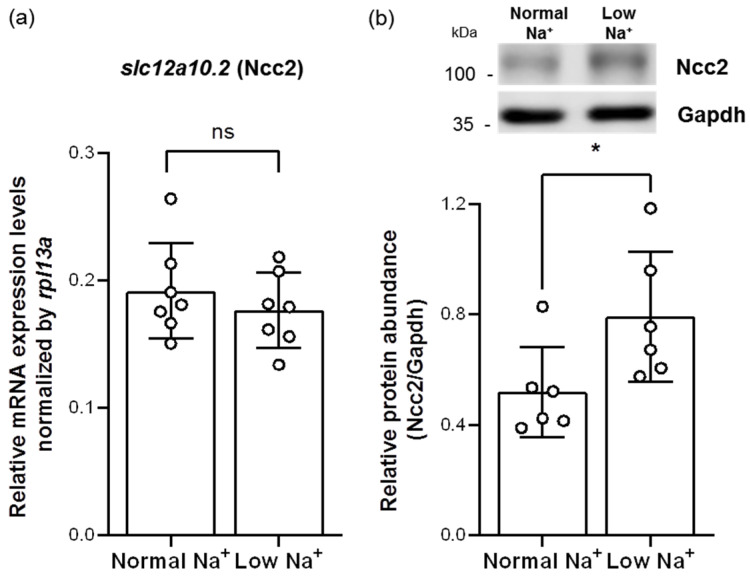
Effects of low-Na^+^ acclimation on Ncc2 expression in the gills of adult zebrafish. The mRNA expression levels of *slc12a10.2* (Ncc2) in the gills were analyzed by quantitative real-time polymerase chain reaction (qRT-PCR) after acclimation to low-Na^+^ FW. qRT-PCR data were normalized to *rpl13a* (N = 7) (**a**). The protein expression of Ncc2 in the gills acclimated to low-Na^+^ FW was analyzed by Western blot. The blots show the bands with molecular weights corresponding to Ncc2 and GAPDH (approximately 112 kDa and 35 kDa, respectively). The protein expression of Ncc2 was quantified and normalized to GAPDH (N = 6) (**b**). Values are the mean ± SD. Student’s *t*-test, * *p* < 0.05. ns, not significant.

To assess whether Ncc2 would be involved in low-Na^+^ acclimation mechanisms, adult zebrafish were treated with long-term exposure of low-Na^+^ FW for 7 days and then subjected to analyses of quantitative real-time polymerase chain reaction (qRT-PCR) and Western blot. Similar to a previous study in tilapia gills [23], low-Na^+^ FW did not affect the mRNA expression of branchial *slc12a10.2* (Ncc2) (Figure 3a). However, we found that the protein expression of Ncc2 was increased in the gills acclimated to low-Na^+^ FW (Figure 3b), suggesting that the NCC ionocyte number and/or the Ncc2 protein expression of individual NCC ionocytes had probably been increased.

### 2.3. Low-Na^+^ FW Increased the Number of NCC Ionocytes and the Ncc2 Protein Expression of Individual NCC Ionocytes in the Gills

**Figure 4:**
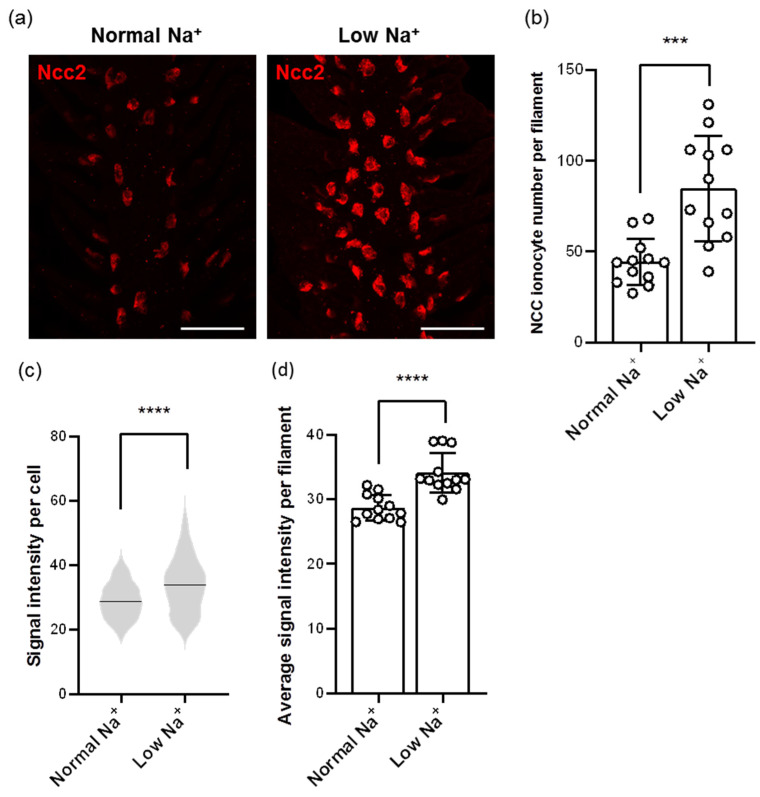
Effects of low-Na^+^ acclimation on the number of NCC ionocytes and Ncc2 expression within individual NCC ionocytes in the gills of adult zebrafish. The cell number of NCC ionocytes and the fluorescence intensity of Ncc2 signals in the gill filaments were analyzed by whole-mount IF after acclimation to low-Na^+^ FW (**a**). NCC ionocyte number of filaments (N = 12) (**b**), mean fluorescence intensity of individual NCC ionocytes (N = 531–1017) (**c**), and the average mean fluorescence intensity of NCC ionocytes of filaments (N = 12) (**d**) are shown. Values are the mean ± SD or SEM. Student’s *t*-test or Mann–Whitney *U* test, *** *p* < 0.001, **** *p* < 0.0001. Scale bar, 50 μm.

Images from whole-mount IF of zebrafish gills using the Ncc2 antibody showed different patterns in the number and signal intensity of NCC ionocytes after low-Na^+^ acclimation (Figure 4a). After calculating with the Imaris software, we found that the number of NCC ionocytes was elevated in low-Na^+^-acclimated gills (Figure 4a,b). In addition, the signal intensity of NCC ionocytes was also increased (Figure 4a,c,d), suggesting that Ncc2 protein expression of individual NCC ionocytes was upregulated.

### 2.4. Low-Na^+^ FW Elevated Branchial Na^+^ Uptake

**Figure 5:**
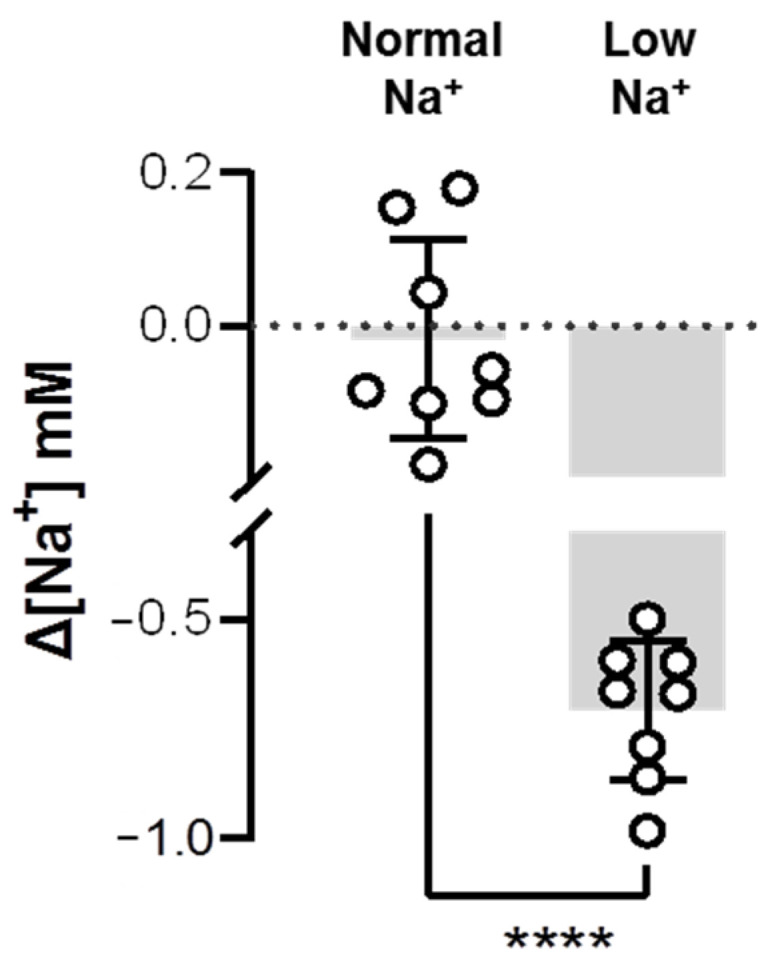
Effects of low-Na^+^ acclimation on branchial Na^+^ uptake capacity of adult zebrafish. The Na^+^ gradient at the gills of adult fish was measured using the scanning ion-selective electrode technique (SIET) after 7 d of acclimation to low-Na^+^ FW. Values are the mean ± SD (N = 8). Student’s *t*-test, **** *p* < 0.0001.

Combined with our findings on upregulation of Ncc2 (Figure 3 and Figure 4) and previous evidence on the upregulation of Nhe3 after low-Na^+^ acclimation [30,31,32], it was expected that Na^+^ uptake would be elevated in the low-Na^+^-acclimated gills of zebrafish. Thus, this study used the SIET to analyze Na^+^ activities between the gills and the background, and calculated the ∆[Na^+^]. As the branchial Na^+^ activity was smaller than the background Na^+^ activity, the calculated ∆[Na^+^] would have a minus value, representing the surrounding Na^+^ taken up by the gills. Indeed, the results showed that low-Na^+^-acclimated gills had a greater ∆[Na^+^] than the normal-Na^+^-acclimated gills (Figure 5), suggesting that branchial Na^+^ uptake ability was enhanced after low-Na^+^ acclimation.

### 2.5. Cl^−^-Free FW Decreased Na^+^ Uptake in the Gills Acclimated to Low-Na^+^ FW

**Figure 6:**
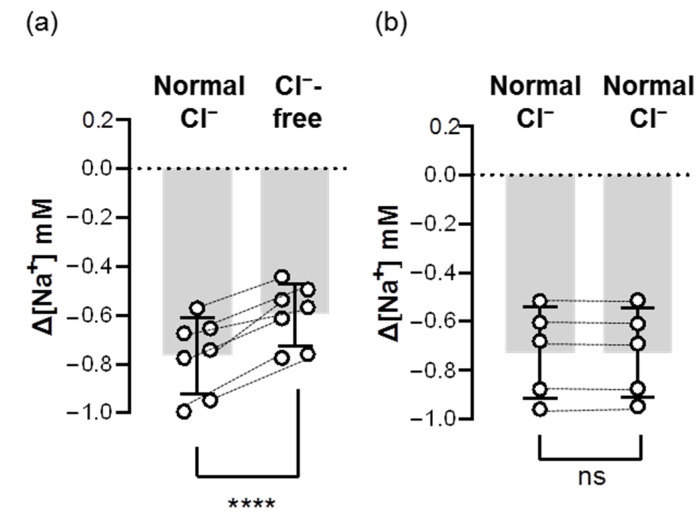
Acute effects of Cl^−^-free FW on the branchial Na^+^ uptake capacity of adult zebrafish. Na^+^ gradients at low-Na^+^-acclimated gills were analyzed before and after the acute incubation of Cl^−^-free (**a**) or normal Cl^−^ (**b**) FW media using the SIET. The dashed line represents the same fish. Values are the mean ± SD (N = 5–7). Student’s *t*-test, **** *p* < 0.0001. ns, not significant.

To test whether Ncc contributes to branchial Na^+^ uptake, we first removed Cl^−^ from FW to block the function of Ncc. We found that acute treatment of Cl^−^-free FW lowered the branchial ∆[Na^+^] by around 20–30% (Figure 6), suggesting that Ncc is involved in Na^+^ uptake in the gills acclimated to low-Na^+^ FW.

### 2.6. Ncc Inhibitor Impaired Na^+^ Uptake in the Gills Acclimated to Low-Na^+^ FW

**Figure 7:**
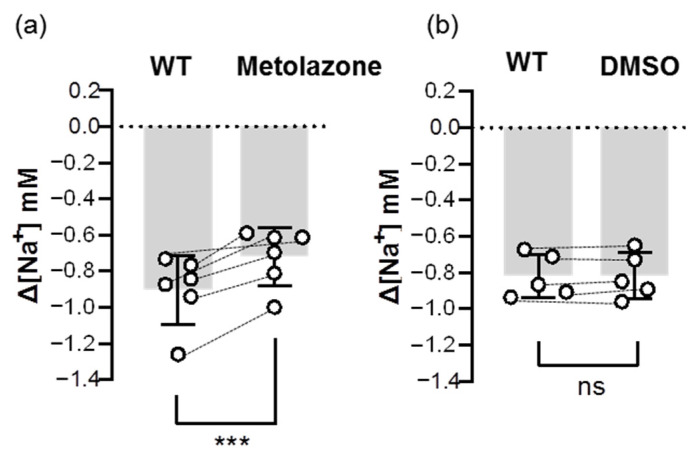
Effects of metolazone on the branchial Na^+^ uptake capacity of adult zebrafish. Na^+^ gradients at low-Na^+^-acclimated gills were analyzed before and after the treatments of metolazone (**a**) or DMSO (**b**) using the SIET. The dashed line represents the same fish. Values are the mean ± SD (N = 5–6). Student’s *t*-test, *** *p* < 0.001. ns, not significant. WT, wild-type.

To further confirm the uptake role of Ncc, we examined the inhibitory effect of an Ncc inhibitor (metolazone) on the Na^+^ uptake function of Ncc. The results showed that the metolazone treatment decreased around 20–30% of ∆[Na^+^] in the gills that had been acclimated to low-Na^+^ FW (Figure 7), suggesting that branchial Ncc does indeed contribute to Na^+^ uptake.

### 2.7. Nhe Inhibitor Impaired Na^+^ Uptake in the Gills Acclimated to Low-Na^+^ FW

**Figure 8:**
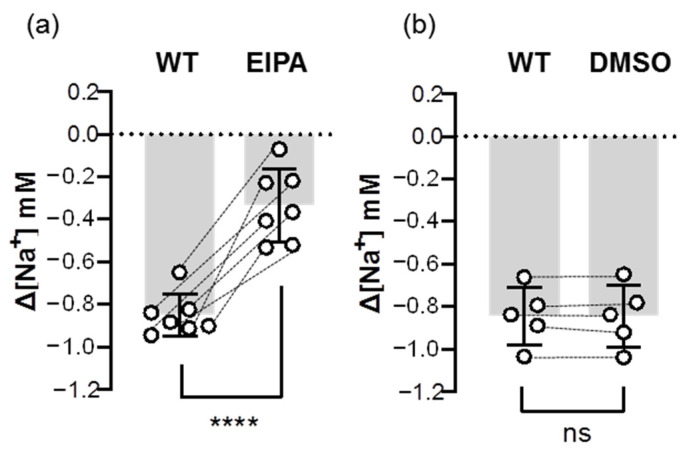
Effects of EIPA on the branchial Na^+^ uptake capacity of adult zebrafish. Na^+^ gradients at low-Na^+^-acclimated gills were analyzed before and after the treatments of EIPA (**a**) or DMSO (**b**) using the SIET. The dashed line represents the same fish. Values are the mean ± SD (N = 5–7). Student’s *t*-test, **** *p* < 0.0001. ns, not significant. EIPA, 5-(N-ethyl-N-isopropyl)-amiloride. WT, wild-type.

In addition to Ncc, we also examined the inhibitory effect of an Nhe inhibitor (EIPA) on the Na^+^ uptake function of Nhe. The results showed that the EIPA treatment decreased over 60% of ∆[Na^+^] in the gills that had been acclimated to low-Na^+^ FW (Figure 8), suggesting that Nhe contributes to the majority of branchial Na^+^ uptake.

## 3. Discussion

The present study applied two powerful analysis techniques (scRNA-Seq and SIET) in zebrafish gills. We clearly identified two subtypes of ionocytes for Na^+^ uptake (NCC ionocytes and Nhe3b-expressing HR ionocytes) and successfully detected branchial Na^+^ activities that are mediated by both Ncc and Nhe. We further demonstrated that acclimation to low-Na^+^ FW increased the contribution of branchial Ncc2 to Na^+^ uptake by upregulating its protein expression and the number of NCC ionocytes. Our findings support and consolidate the role of Ncc in the branchial Na^+^ uptake of adult FW teleosts, and also shed light on the ionoregulatory mechanisms in gill physiology.

As the two ions co-transported by Ncc, Na^+^ and Cl^−^ have been reported to affect the functional regulation of Ncc. In the tubule epithelial cells of mammalian kidneys, the protein activity and uptake function of Ncc are regulated by intracellular Cl^−^ concentration, which is mediated by post-translational modification via the WNK-SPAK signaling pathways [33,34]. In rodents, the amount of NaCl in the diet affects renal Ncc protein expression and/or its activity [35,36]. Similarly, in several FW teleost species, the expression and uptake function of Ncc in the skin or gill ionocytes could be triggered by low-Cl^−^ FW [18,21,23,37]. New findings from the present study showed that low-Na^+^ FW triggered Ncc2 protein expression in each branchial NCC ionocyte (Figure 4a,c) without affecting the mRNA expression in zebrafish gills (Figure 3a), hinting at the existence of an undiscovered mechanism of translational regulation. This is probably the reason why previous studies that only analyzed mRNA expression could not clarify whether teleost Ncc was important in low-Na^+^ acclimation [18,21,23]. Low-Na^+^ FW also increased the number of NCC ionocytes in the gills (Figure 4a,b), implying that stem cell proliferation and/or ionocyte differentiation probably occurred. This phenomenon of an increased NCC ionocyte number could also be observed in the larval skin of low-Na^+^-acclimated zebrafish (Figure S4). That is to say, the NCC ionocyte number and Ncc protein expression are important indicators that should be taken into consideration when studying teleost Ncc regulation. In short, Na^+^, in addition to Cl^−^, is also a critical factor for determining Ncc regulation in FW teleosts, and this regulation would be positively triggered when there is a shortage of environmental Na^+^.

Lacking salt would induce physiological compensation for body-fluid Na^+^ homeostasis such as in the case of changing the ion and water retention capacities of mammalian nephrons. In order to lower the final loss of Na^+^ in the urine and keep the blood Na^+^ concentration within a normal physiological range, mammals fed a low-Na^+^ diet would decrease their glomerular filtration rate (GFR) and increase Na^+^ reabsorption of the nephrons by upregulating the expression of renal Ncc and epithelial Na^+^ channel (Enac), but not necessarily Nhe3 [38,39]. Notably, in teleosts (lacking Enac genes [40]), a long-term exposure to low-Na^+^ FW would trigger both Ncc and Nhe3, and thereby enhance Na^+^ uptake capacity (Figure 3, Figure 4 and Figure 5) [30,31,32,41,42]. In fact, it is reasonable to observe compensatory mechanisms of discrepancy between mammals and teleosts in the regulation of Nhe3 and Ncc. Functionally similar but facing different external media, mammalian renal epithelial cells are located in the lining of the lumen with a high osmolarity fluid ([Na^+^] = 25–350 mM [43,44]), but teleost ionocytes are exposed to hypotonic FW ([Na^+^] < 1 mM [9,41]). In addition, filtered Na^+^ from the glomeruli is sequentially absorbed along the tubule of nephrons, first by Nhe3 and then by Ncc and Enac [45]. However, the distribution of Nhe3- and Ncc-expressing ionocytes is likely interlaced on the skin/gill surfaces (Figure 2 and Figure S2) [16,18,21,23], so they presumably take up Na^+^ from FW simultaneously.

Interestingly, in spite of the difference between the mammals and teleosts above-mentioned, the contribution degree of Nhe3 and Ncc to Na^+^ absorption was similar in the renal epithelial cells and gill ionocytes. In mammalian nephrons, different segments exhibit a functional division for Na^+^ reabsorption. The proximal convoluted tubule (PCT, where the Nhe3 expresses) accounts for about 60% of the total Na^+^ reabsorption. Although some Na^+^-coupled co-transporters (e.g., Na^+^-glucose co-transporters) are also expressed in PCT, the contribution of them to Na^+^ reabsorption is small, which suggests that Nhe3 presumably plays a dominant role in PCT Na^+^ reabsorption [45]. The distal convoluted tubule (DCT, where the Ncc expresses) and collecting duct (CD, where the Enac expresses) account for less than 10% of the total Na^+^ reabsorption [45]. In teleosts, electrophysiological and pharmacological studies in the larval skin suggest that around 70% and 75% of Na^+^ is taken up by Nhe in medaka and in low-Na^+^-acclimated zebrafish, respectively [30,41]. Adult zebrafish treated with EIPA (Nhe inhibitor) also showed an over 50% decline in whole-body Na^+^ influx (according to measurements made with a radiotracer) [7]. These suggest that the contribution of Nhe to Na^+^ uptake is more than that of Ncc. However, radioisotope experiments in adult zebrafish unexpectedly showed that neither metolazone (Ncc inhibitor) nor a Cl^−^-free medium reduced the whole-body Na^+^ influxes [7]. These findings against Ncc’s role in Na^+^ uptake are unexplainable [7] because it is well-known that omitting Cl^−^ from the medium is a powerful way to impair the Na^+^ uptake function of Ncc [46,47,48,49]; besides, metolazone has also been proven to be an effective inhibitor that attenuates the functions of Ncc in several species including rat, flounder, tilapia, and even zebrafish [21,37,46,47], although eel Ncc is resistant to this inhibitor [48,49]. Here, the present study used adult zebrafish to clarify and confirm the effectiveness of metolazone and Cl^−^-free treatments by directly analyzing the Na^+^ uptake function of the gills and recording the change in the Na^+^ uptake in the same fish before and after treatments. Both the Cl^−^-free FW and metolazone impaired branchial Na^+^ uptake of the gills in adult zebrafish (Figure 6 and Figure 7). Of note, we demonstrated that around 20–30% and 60% of Na^+^ were taken up by Ncc and Nhe, respectively, in the gills of low-Na^+^-acclimated zebrafish (Figure 7 and Figure 8). These findings and the comparisons between mammals and teleosts raise the question of why teleosts tend to utilize Nhe (instead of Ncc) as the major transporter for Na^+^ uptake even though the environmental conditions and the cell distribution are totally different from those in mammalian kidneys. A possible reason is that ionocytes contain a higher intracellular [NH_4_^+^] than FW [9,41]. Because Nhe exhibits not only Na^+^/H^+^ but Na^+^/NH_4_^+^ exchange activities, teleost ionocytes most likely rely on a favorable outward NH_4_^+^ gradient to efficiently drive Nhe and simultaneously take up Na^+^ from hypotonic FW [9,10,41,50]. However, the electrical/chemical gradients generated by basolateral Nka seem to be ineffective in driving ionocyte Ncc against unfavorable Na^+^/Cl^−^ gradients [3,9]. Although an idea of Nka isoform switching has been proposed [51], whether and how Ncc is driven by Nka (and/or probably other unknown transporters) should be further investigated.

Basolateral Nka, comprising multiple α and β subunits, is a critical enzyme for body fluid ionic and osmotic homeostasis and other physiological processes. In our scRNA-Seq-based identification of gill cells, we further found that three subtypes of ionocytes expressed different Nka α1 subunits (Figure 1 and Figure S1), and renewed the previous ionocyte model of zebrafish at α1 subunits [17,26]. Indeed, the expression distribution of the α subunit isoforms is thought to be associated with the functions of specific cells or tissues, since the enzyme kinetics of Nka is determined by α subunits [26,52]. That is, our findings link a possible connection between different combinations of Nka subunits and the ion-transporting functions of distinct ionocytes. Combined with further functional analyses, we may reveal how teleost Ncc is driven in future studies.

In addition to the two main transporters (Nhe and Ncc) for teleost Na^+^ uptake, we previously mentioned another proposed model for Na^+^ uptake, the apical VHa-driven Asic-mediated pathway [4,5,6,7], in the introduction. According to previous observation of an inhibition of Na^+^ uptake by treating with bafilomycin (a VHa inhibitor) in FW tilapia, carp, zebrafish, and trout [20,53,54], the VHa-driven Asic model was considered to play a potential role in electrogenic Na^+^ uptake via Asic for a long time [4,5,6]. However, only a few FW teleosts (e.g., zebrafish) express VHa in the apical membrane of ionocytes [10], and Asic was only reported to be expressed in zebrafish and trout ionocytes by IF staining (without identification by in situ hybridization) [4,5,6]. These suggest that this proposed model cannot fit most FW teleosts. In addition, we accidentally found that Asic was not expressed in the ionocytes of zebrafish gills by scRNA-Seq analysis (Figure S5), suggesting that Asic expression is actually very low and the importance of Asic to Na^+^ uptake is questionable. By the way, we also could not find any other Na^+^ channel expressed in gill ionocytes of zebrafish from our scRNA-Seq data. On the other hand, bafilomycin treatment is widely-known to greatly alter cellular processes [55,56,57,58] because VHa contributes to vesicular/protein trafficking, recycling, endocytosis, protein degradation, autophagy, and cell signaling [58]. The inhibition of teleost Na^+^ uptake by bafilomycin [20,53,54] is probably the effect of VHa assembly disruption on cellular homeostasis. A recent study in zebrafish further reinforced this notion and demonstrated that bafilomycin would disrupt membrane protein sorting and trafficking [55]. Use of bafilomycin for examining Na^+^ uptake may not prove the coupling function of VHa and Asic (or other Na^+^ channel). Most importantly, the gating property of Asic keeps itself inactive but only “transiently” open upon certain situations (acidified medium) [12,13]; that is, it seems improbable that the external Na^+^ could be taken by Asic under FW [11]. This is probably why there are inconsistent results from inhibitor and knockdown experiments by previous studies that Asic inhibitors can impair whole-body Na^+^ influxes of juvenile trout and adult zebrafish [5,6], but this uptake function could not be reduced by the same inhibitors or Asic4b knockdown in larval zebrafish [4]. As such, the VHa-driven Asic model in teleosts remains largely controversial and can be eliminated [9].

In summary, the present study used scRNA-Seq and triple IF to clearly identify that Nhe3b-expressing HR ionocytes, NaR ionocytes, and NCC ionocytes are the three distinct subtypes of ionocytes in the gills of zebrafish. We found that teleosts may not regulate the mRNA expression of Ncc, but they do trigger its protein expression and NCC ionocyte number when coping with low-Na^+^ FW environments, suggesting a translational control of teleost Ncc and cell proliferation/differentiation. Most importantly, for the first time, we performed in vivo functional assays to prove the uptake functions of Nhe and Ncc directly in the gills. This study strengthened the claim that zebrafish gills utilize both Nhe-mediated and Ncc-mediated pathways to take up Na^+^ from FW, which clarify the debates on the roles of both Nhe and Ncc, and also broadens our understanding of branchial Na^+^ regulation in fish physiology.

## 4. Materials and Methods

### 4.1. Experimental Animals

Adult zebrafish (*Danio rerio*) were kept in local tap FW at 28 °C under a 14:10-h light–dark photoperiod at the Institute of Cellular and Organismic Biology (ICOB), Academia Sinica, Taipei City, Taiwan. The experimental protocols were designed following the Academia Sinica Institutional Animal Care and Use Committee (approval no.: 17-12-1163). Before sacrifice or removal of the opercula, zebrafish were anesthetized with 0.3 mg/L ethyl 3-aminobenzoate (MS-222) (dissolved in FW). To detect the Na^+^ activities in the gills, the opercula were cut in a certain proportion before the SIET detection.

### 4.2. Single-Cell RNA Sequencing (scRNA-Seq)

Sample collection and library establishment for scRNA-Seq were performed following our recent publication [59]. The results of the raw reads were demultiplexed and aligned to the *Danio rerio* genome (Release 103) from the NCBI, following the guidance of the Cell Ranger software (10× Genomics, version 6.1.1). The output showed 8952 successfully tagged and sequenced cells. The analysis finally resulted in 2265 median reads and 453 median genes per cell. Data were then browsed and inspected in the Loupe browser (10× Genomics, version 6).

### 4.3. Low-Na^+^ Acclimation Experiment

The artificial normal- and low-Na^+^ FWs were prepared by adding adequate amounts of Na_2_SO_4_, KH_2_PO_4_, K_2_HPO_4_, CaSO_4_·2H_2_O, MgSO_4_, and MgCl_2_·6H_2_O to aerated deionized water, and the ion levels of the media are shown in Table S1. Adult zebrafish were acclimated to normal- or low-Na^+^ FW for 7 d without feeding. The medium was changed daily during the acclimation experiments to maintain water quality. No mortality was observed during the acclimation period.

### 4.4. Preparation of Complementary DNA

Zebrafish gills excised from one individual were pooled as one sample and homogenized in TRIzol Reagent (Invitrogen, Waltham, MA, USA). Following the protocol from the manufacturer, the total RNA of the gills was extracted. Removal of the genomic DNA was achieved by treating with DNase I (Roche, Basel, Switzerland). Using a NanoDrop 2000 (Thermo Scientific, Waltham, MA, USA), the quality and quantity of the total RNA were checked and calculated. Following the manufacturer’s protocol, 2.5 μg of the total RNA was used to synthesize complementary DNA with SuperScript IV reverse transcriptase (Thermo Scientific, Waltham, MA, USA). After synthesis, complementary DNA was diluted with sterile deionized water and stored at −20 °C until use.

### 4.5. Quantitative Real-time Polymerase Chain Reaction (qRT-PCR)

A Light Cycler real-time PCR system (Roche, Basel, Switzerland) was used to perform qRT-PCR following a previous protocol [60]. Ribosomal protein L13a (*rpl13a*) was selected as an internal control for zebrafish. The sequences and amplification efficiency of each primer set are shown in Table S2. The specificity of each primer set was confirmed by Sanger sequencing of the amplicons.

### 4.6. Immunofluorescence (IF)

The gills (excised from four individuals) and six larvae (4 days post-fertilization, dpf) were fixed with 4% paraformaldehyde in phosphate-buffered saline (PBS). Then, whole-mount IF was conducted following a previous study [22]. Custom rabbit Ncc2 polyclonal antibody against the N-terminal domain (IKKSRPSLDVLRNPPDD) of zebrafish Ncc2 (2 µg/mL) [22], a CoraLite^®^647-conjugated rabbit Atp6v1a polyclonal antibody (described below) (1 µg/mL), a mouse Nka alpha subunit monoclonal antibody (α5, Developmental Studies Hybridoma Bank, Iowa City, IA, USA) (1:200 dilution), an Alexa Fluor 488 goat anti-mouse immunoglobulin G (IgG) (Invitrogen, Waltham, MA, USA) (1:200 dilution), and an Alexa Fluor 568 goat anti-rabbit IgG (Invitrogen, Waltham, MA, USA) (1:200 dilution) were used. The FlexAble CoraLite^®^ Plus 647 Kit and rabbit Atp6v1a polyclonal antibody, both provided by Proteintech (Rosemont, IL, USA), were utilized to prepare a CoraLite^®^647-conjugated rabbit Atp6v1a polyclonal antibody for labeling.

For the cell counting and fluorescence intensity analysis experiments, the middle parts of the filaments within the third gill pairs in adult zebrafish were chosen for imaging. LSM980 (Zeiss, Jena, Germany) was used to obtain the images within a given length (400 μm in the distal edge of the filament) from three randomly-selected filaments in each adult individual and within the whole skin of each 4 dpf larva. Imaris 9.9 (Oxford Instruments plc, Abingdon, UK) or Fiji [61] were then used to calculate the number and/or fluorescence intensity of the Ncc2-positive cells (NCC ionocytes). For the peptide blocking experiments, 2 µg of custom rabbit Ncc2 polyclonal antibody was mixed with 20 µg of synthetic Ncc2 peptide (IKKSRPSLDVLRNPPDD) in 1 mL PBS and incubated for 2 days before use for IF.

### 4.7. Western Blot

The gills excised from one individual were pooled as one sample for total protein extraction. Protein extraction and Western blot were performed following a previous protocol [62]. A total of 20 μg of total protein was loaded for Western blot. Custom rabbit Ncc2 polyclonal antibody against the N-terminal domain (IKKSRPSLDVLRNPPDD) of zebrafish Ncc2 (400 ng/mL) [22], the GAPDH antibody (GeneTex, Hsinchu City, Taiwan) (1:5000 dilution), and Goat anti-Rabbit IgG (H+L) Secondary Antibody, HRP (Invitrogen, Waltham, MA, USA) (1:5000 dilution) were used for immunoblotting. WesternBright ECL (Advansta, San Jose, CA, USA) was used to produce signals. All images were obtained by a UVP ChemStudio PLUS Imaging System (Analytik Jena, Jena, Germany) and quantitated by Fiji [61].

### 4.8. Scanning Ion-Selective Electrode Technique (SIET)

The SIET was conducted in an agar chamber filled with FW-recording medium (0.25 mM Na_2_SO_4_, 0.16 mM KH_2_PO_4_, 0.16 mM K_2_HPO_4_, 0.2 mM CaSO_4_·2H_2_O, 0.1 mM MgSO_4_, 0.25 mM MgCl_2_·6H_2_O, 0.3 mg/L ethyl 3-aminobenzoate, 300 µM MOPS buffer, pH 7.0) at 26–28 °C. After 3-min anesthesia, zebrafish were properly placed in the chamber [62]. To measure the Na^+^ gradients (represented as ∆[Na^+^]) between the gills and background, a Na^+^-selective microelectrode was generated to detect the Na^+^ activities with ASET software, following the previous studies [42,63]. We first recorded the background Na^+^ activities of the FW-recording medium before the zebrafish were put in the chamber. After the fish were laid out in the chamber, the Na^+^ activities at the interspace of the filaments of the third gill pair (the middle part of the gills) were detected and recorded. Then, the branchial ∆[Na^+^] was calculated.

For the experiments of acute treatments by Cl^−^-free FW, the original Na^+^ activities of the gills of low-Na^+^-acclimated fish were first recorded in the FW-recording medium. Then, we immediately transferred the fish to the Cl^−^-free FW-recording medium (0.25 mM Na_2_SO_4_, 0.16 mM KH_2_PO_4_, 0.16 mM K_2_HPO_4_, 0.2 mM CaSO_4_·2H_2_O, 0.1 mM MgSO_4_, 0.3 mg/L ethyl 3-aminobenzoate, 300 µM MOPS buffer, pH 7.0) and recorded the Na^+^ activities of the gills again. Background Na^+^ activities of the Cl^−^-free FW-recording medium were also detected before each transfer of fish to the Cl^−^-free FW-recording medium. Removal of MgCl_2_ from the FW-recording medium did not change the selectivity and sensitivity of the Na^+^-selective microelectrode.

### 4.9. Pharmacological Treatments

The stocks of 50 mM metolazone (Sigma-Aldrich, Taipei City, Taiwan) and 400 mM 5-(N-ethyl-N-isopropyl)-amiloride (EIPA) (Sigma-Aldrich, Taipei City, Taiwan) were dissolved and prepared in dimethyl sulfoxide (DMSO). The original Na^+^ activities of the gills of low-Na^+^-acclimated fish were recorded in the FW-recording medium, and then the zebrafish were subjected to the treatments of metolazone or EIPA to examine the inhibitory effects on branchial Na^+^ uptake. After treating with inhibitors, Na^+^ activities in the gills of the same zebrafish were then recorded in the FW-recording medium (without containing inhibitors). For the treatment of metolazone, zebrafish were incubated in low-Na^+^ FW containing 100 μM metolazone for 2 h. For the treatment of EIPA, zebrafish were incubated in low-Na^+^ FW containing 100 μM EIPA for 1 h. The concentrations of metolazone and EIPA were selected based on previous studies [7,62].

### 4.10. Statistical Analysis

We first applied a Shapiro–Wilk normality test to assess the normality of all datasets. For the parametric data, the datasets were analyzed using a Student’s *t*-test, and the values are presented as the mean ± standard deviation (SD). For the nonparametric data, the datasets were analyzed using a Mann–Whitney *U* test, and the values are presented as the mean ± standard error of the mean (SEM). Prism 8.4.2 (GraphPad, CA, USA) was used to perform the statistical analyses.

## Data Availability

The raw data used for all statistical analyses can be found at https://drive.google.com/drive/folders/1h3Tbvf8blu7h8cRhjjj-JkndRJYPurKB?usp=share_link, accessed on 31 January 2023.

## Supplementary Materials

The supporting information can be downloaded at: https://www.mdpi.com/article/10.3390/ijms24076597/s1.

## Abbreviations

Asic	Acid-sensing ion channel
CD	Collecting duct
DCT	Distal convoluted tubule
dpf	Day(s) post-fertilization
Ecac	Epithelial Ca^2+^ channel
EIPA	5-(N-ethyl-N-isopropyl)-amiloride
Enac	Epithelial Na^+^ channel
FW	Freshwater
GFR	Glomerular filtration rate
HR	VHa-rich
IF	Immunofluorescence
KO	Knockout
NaR	Nka-rich
Ncc	Na^+^-Cl^−^ co-transporter
Nhe	Na^+^/H^+^ exchanger
Nka	Na^+^/K^+^ ATPase
PCT	Proximal convoluted tubule
qRT-PCR	Quantitative real-time polymerase chain reaction
scRNA-Seq	Single-cell RNA sequencing
SIET	Scanning ion-selective electrode technique
SW	Seawater
VHa	Vascular-type H^+^ ATPase
