# Supplementary material for: Molecular Physiological Evidence for the Role of Na+-Cl− Co-Transporter in Branchial Na+ Uptake in Freshwater Teleosts
Source: Int J Mol Sci. 2023 Apr 1;24(7):6597. doi: 10.3390/ijms24076597 (PMC10094795; doi:10.3390/ijms24076597)
Supplement: Supplementary file 1 [file ijms-24-06597-s001.zip › ijms-2221450-supplementary/Figure S1.pdf]

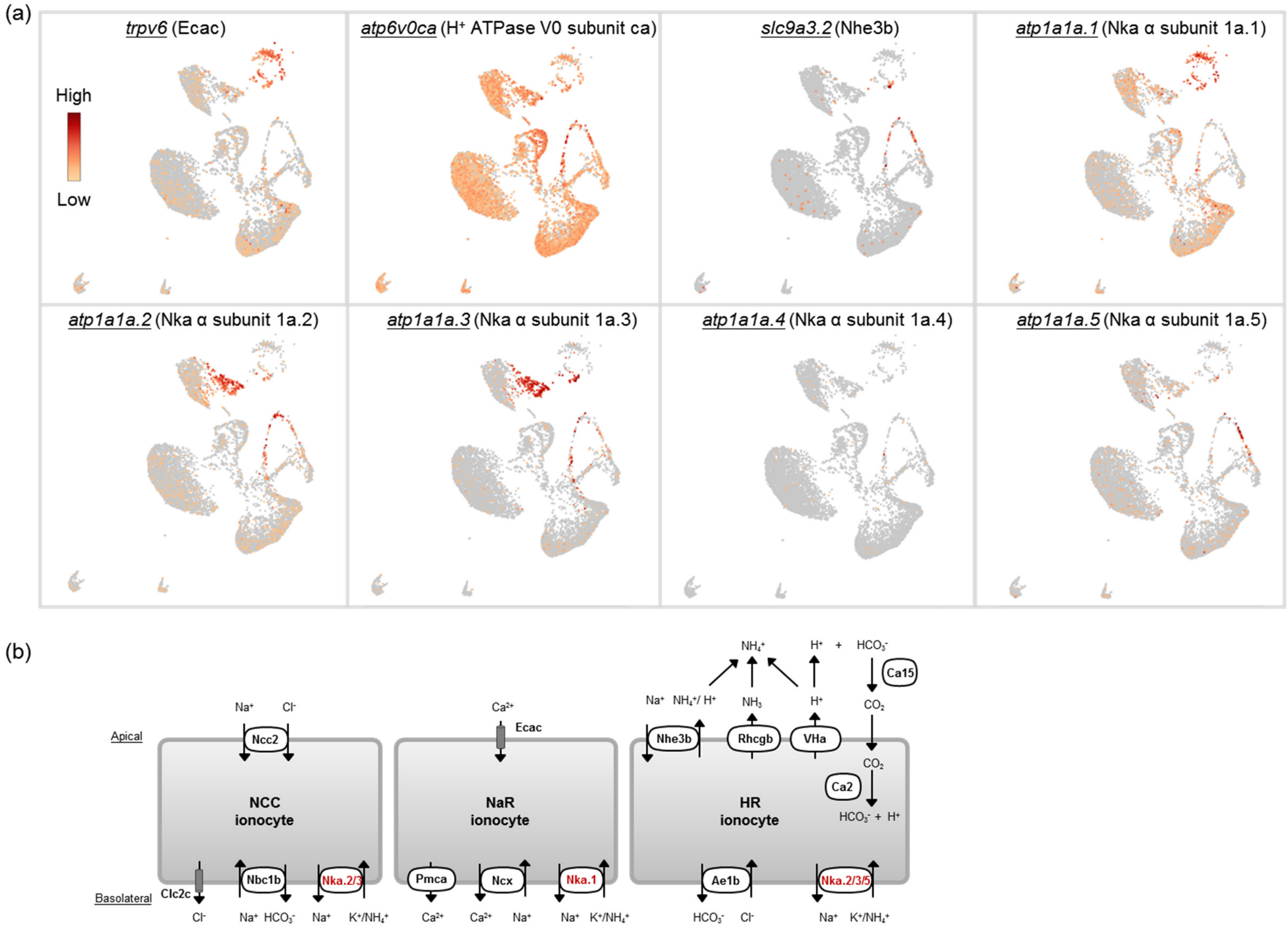

Figure S1. The expression of selected genes on the UMAP and newly-proposed ionocyte model in the gill cells of adult zebrafish. The UMAP shows the distribution and expression of *trpv6* (epithelial Ca^{2+} channel, Ecac), *atp6v0ca* (H^+ ATPase V0 subunit ca), *slc9a3.2* (Na^+/H^+ exchanger 3b, Nhe3b), *atp1a1a.1* (Nka *α* subunit 1a.1), *atp1a1a.2* (Nka *α* subunit 1a.2), *atp1a1a.3* (Nka *α* subunit 1a.3), *atp1a1a.4* (Nka *α* subunit 1a.4), and *atp1a1a.5* (Nka *α* subunit 1a.5) in zebrafish gill cells (a). Newly-proposed ionocyte model of zebrafish is shown (b), which summarizes the findings in (a). Ae1b, anion exchanger 1b; Ca2, cytosolic carbonic anhydrase 2; Ca15, membrane-bound carbonic anhydrase 15; Clc2c, chloride channel 2c; Nbc1b, Na^+/HCO_3^- co-transporter 1b; Ncc2, Na^+-Cl^- co-transporter 2; Ncx, Na^+/Ca^{2+} exchanger; Nka.1~.5, Nka *α* subunit 1a.1~1a.5; Pmca, plasma membrane Ca^{2+} ATPase; Rhcgb, rhesus C glycoprotein b; VHa, vascular-type H^+ ATPase.
