# Supplementary material for: Molecular Physiological Evidence for the Role of Na+-Cl− Co-Transporter in Branchial Na+ Uptake in Freshwater Teleosts
Source: Int J Mol Sci. 2023 Apr 1;24(7):6597. doi: 10.3390/ijms24076597 (PMC10094795; doi:10.3390/ijms24076597)
Supplement: Supplementary file 1 [file ijms-24-06597-s001.zip › ijms-2221450-supplementary/Figure S2.pdf]

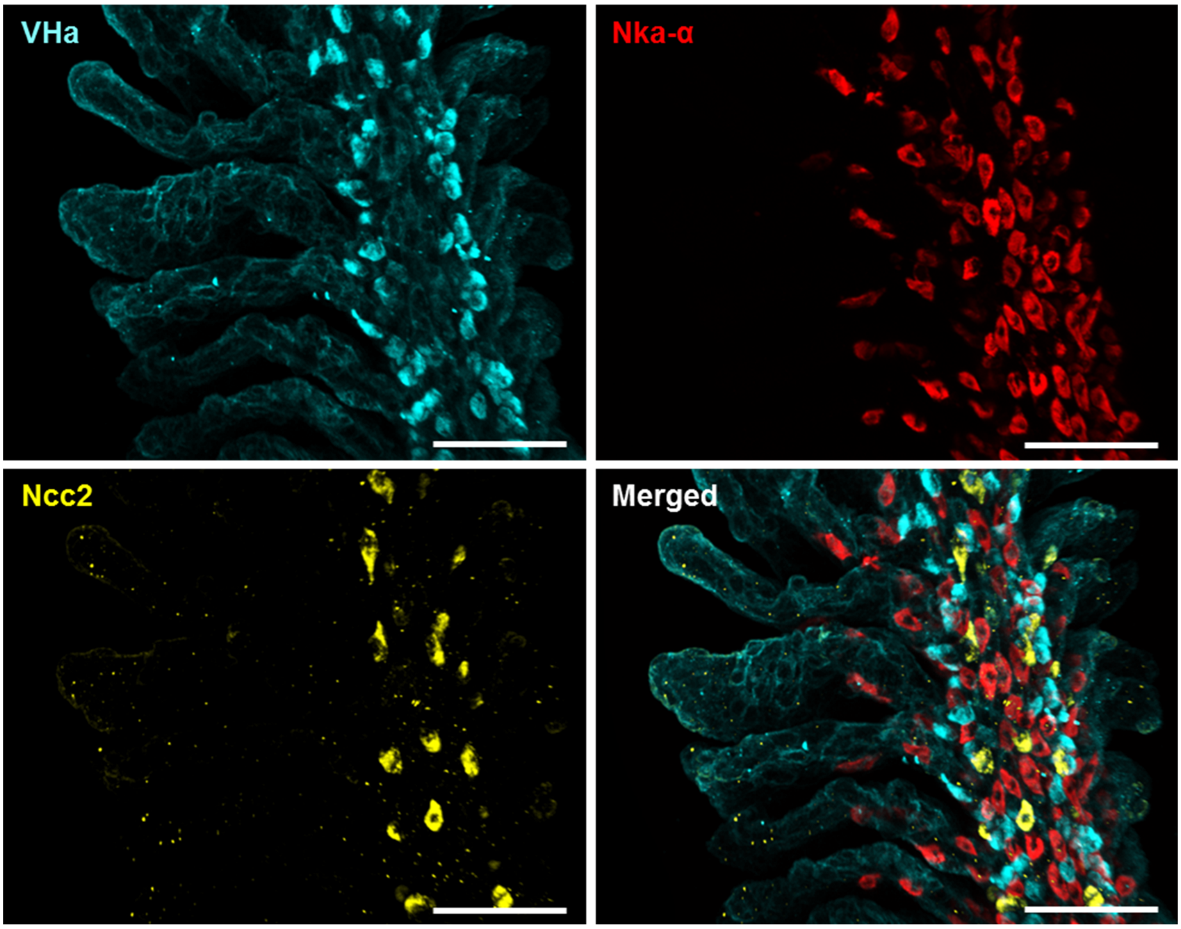

Figure S2. Localization of three subtypes of ionocytes in the gills of adult zebrafish. A different angle of Figure 2 is shown. Scale bar, 50 μm.
