# Supplementary material for: Molecular Physiological Evidence for the Role of Na+-Cl− Co-Transporter in Branchial Na+ Uptake in Freshwater Teleosts
Source: Int J Mol Sci. 2023 Apr 1;24(7):6597. doi: 10.3390/ijms24076597 (PMC10094795; doi:10.3390/ijms24076597)
Supplement: Supplementary file 1 [file ijms-24-06597-s001.zip › ijms-2221450-supplementary/Figure S3.pdf]

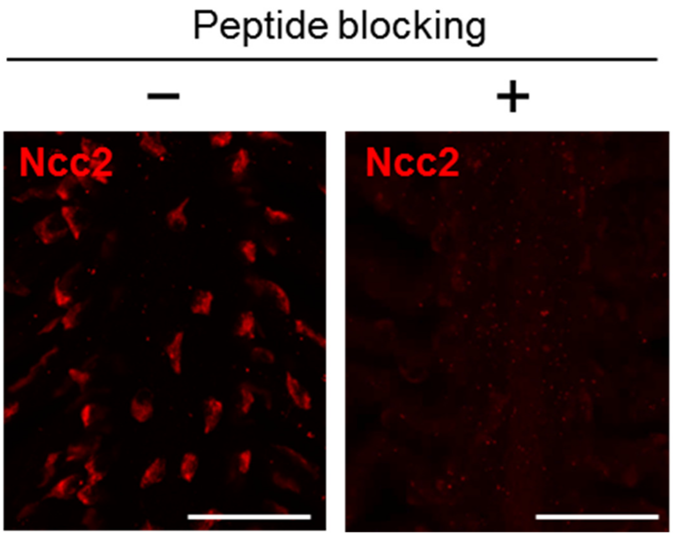

Figure S3. Examination of the specificity of Ncc2 antibody in zebrafish gills. The gills stained by Ncc2 antibody pre-incubated with (+) or without (−) synthetic Ncc2 peptide were shown by whole-mount IF. Scale bar, 50 μm.
