# Supplementary material for: Molecular Physiological Evidence for the Role of Na+-Cl− Co-Transporter in Branchial Na+ Uptake in Freshwater Teleosts
Source: Int J Mol Sci. 2023 Apr 1;24(7):6597. doi: 10.3390/ijms24076597 (PMC10094795; doi:10.3390/ijms24076597)
Supplement: Supplementary file 1 [file ijms-24-06597-s001.zip › ijms-2221450-supplementary/Figure S4.pdf]

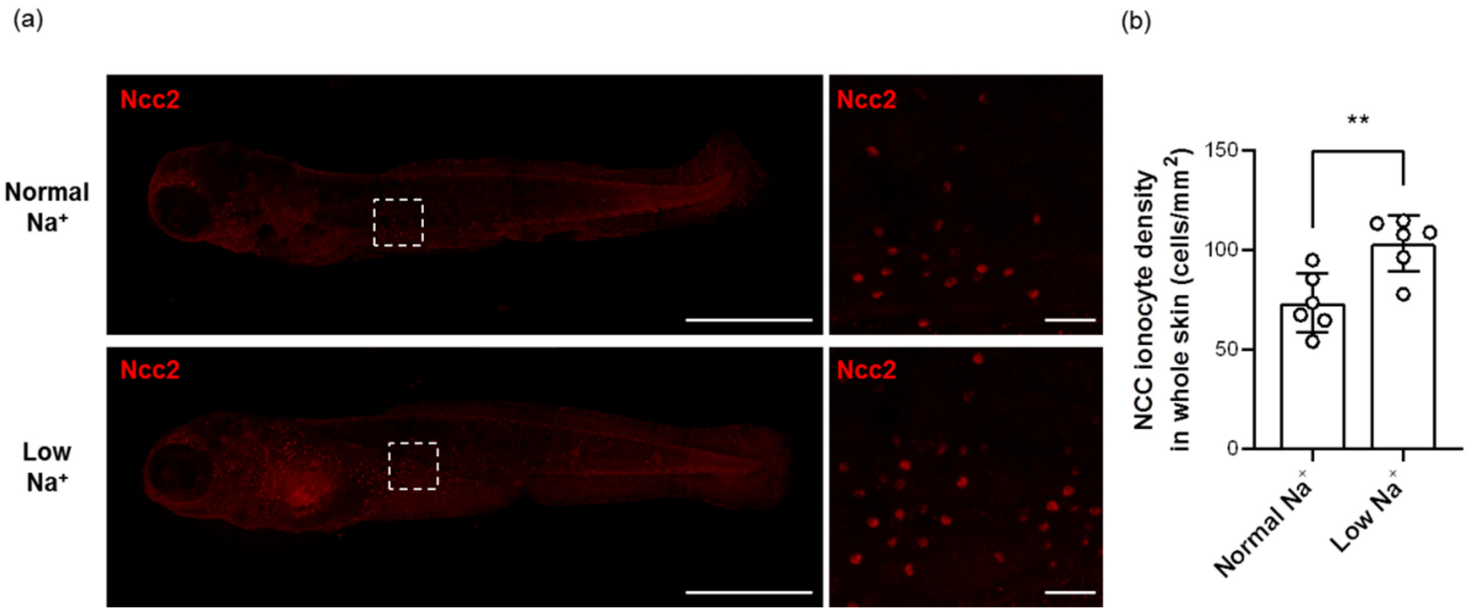

Figure S4. Effects of low-Na$^+$ acclimation on the number of NCC ionocytes in the skin of 4 days post-fertilization (dpf) larval zebrafish. After acclimation to low-Na$^+$ FW, NCC ionocytes in the whole skin of 4 dpf larvae were stained using IF. The right panels (scale bar, 50 µm) show enlargements of the indicated areas (white dashed frames) in the left panels (scale bar, 500 µm) (a). Cell density of NCC ionocytes was calculated and shown (b). Values are the mean ± SD (N=6). Student's *t*-test, ** $p < 0.01$.
