# Supplementary material for: Molecular Physiological Evidence for the Role of Na+-Cl− Co-Transporter in Branchial Na+ Uptake in Freshwater Teleosts
Source: Int J Mol Sci. 2023 Apr 1;24(7):6597. doi: 10.3390/ijms24076597 (PMC10094795; doi:10.3390/ijms24076597)
Supplement: Supplementary file 1 [file ijms-24-06597-s001.zip › ijms-2221450-supplementary/Figure S5.pdf]

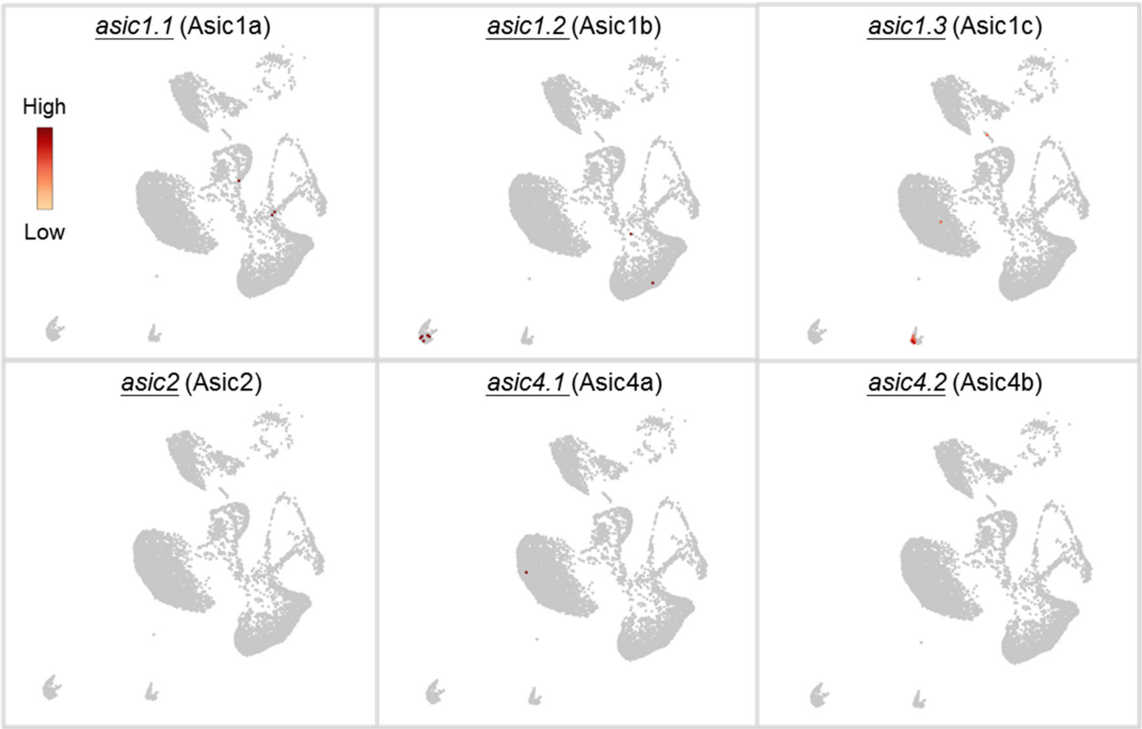

Figure S5. The expression of paralogs of *asic* on the UMAP in the gills of adult zebrafish. The UMAP shows the distribution and expression of *asic1.1* (Acid-sensing ion channel 1a, Asic1a), *asic1.2* (Asic1b), *asic1.3* (Asic1c), *asic2* (Asic2), *asic4.1* (Asic4a), and *asic4.2* (Asic4b) in gill cells of adult zebrafish.
