# Supplementary material for: Molecular Physiological Evidence for the Role of Na+-Cl− Co-Transporter in Branchial Na+ Uptake in Freshwater Teleosts
Source: Int J Mol Sci. 2023 Apr 1;24(7):6597. doi: 10.3390/ijms24076597 (PMC10094795; doi:10.3390/ijms24076597)
Supplement: Supplementary file 1 [file ijms-24-06597-s001.zip › ijms-2221450-supplementary/Table S1.pdf]

Table S1. Ion concentration of artificial normal- and low-Na^+ FW.

	Na^+ (mM)	Cl^- (mM)	K^+ (mM)	Mg^{2+} (mM)	Ca^{2+} (mM)
Normal-Na^+ FW	0.5	0.2	0.24	0.2	0.2
Low-Na^+ FW	0.001	0.2	0.24	0.2	0.2
