# Supplementary material for: Molecular Physiological Evidence for the Role of Na+-Cl− Co-Transporter in Branchial Na+ Uptake in Freshwater Teleosts
Source: Int J Mol Sci. 2023 Apr 1;24(7):6597. doi: 10.3390/ijms24076597 (PMC10094795; doi:10.3390/ijms24076597)
Supplement: Supplementary file 1 [file ijms-24-06597-s001.zip › ijms-2221450-supplementary/Table S2.pdf]

Table S2. Primer sets used for quantitative real-time polymerase chain reaction (qRT-PCR).

Gene name	Accession number		Sequence (5' to 3')	Primer efficiency
rpl13a	NM_212784	Forward<br>Reverse	CCTCGGTCGTCTTTCCGCTATTG<br>CAGCCTGACCCCTCTTGGTTTTG	95.2%
slc12a10.2	NM_001045001	Forward<br>Reverse	GCCCCCAAAGTTTTCCAGTT<br>TAAGCACGAAGAGGCTCCTTG	95.9%
